# The crucial role of the regulatory mechanism of the Atg1/ULK1 complex in fungi

**DOI:** 10.3389/fmicb.2022.1019543

**Published:** 2022-10-26

**Authors:** Ying-Ying Cai, Lin Li, Xue-Ming Zhu, Jian-Ping Lu, Xiao-Hong Liu, Fu-Cheng Lin

**Affiliations:** ^1^State Key Laboratory for Managing Biotic and Chemical Treats to the Quality and Safety of Agro-products, Institute of Plant Protection and Microbiology, Zhejiang Academy of Agricultural Sciences, Hangzhou, China; ^2^State Key Laboratory for Managing Biotic and Chemical Treats to the Quality and Safety of Agro-products, Institute of Biotechnology, Zhejiang University, Hangzhou, China; ^3^College of Life Science, Zhejiang University, Hangzhou, China

**Keywords:** Atg1/ULK1 complex, autophagy, TOR, AMPK/Snf1, regulatory mechanism

## Abstract

Autophagy, an evolutionarily conserved cellular degradation pathway in eukaryotes, is hierarchically regulated by autophagy-related genes (Atgs). The Atg1/ULK1 complex is the most upstream factor involved in autophagy initiation. Here，we summarize the recent studies on the structure and molecular mechanism of the Atg1/ULK1 complex in autophagy initiation, with a special focus on upstream regulation and downstream effectors of Atg1/ULK1. The roles of pathogenicity and autophagy aspects in Atg1/ULK1 complexes of various pathogenic hosts, including plants, insects, and humans, are also discussed in this work based on recent research findings. We establish a framework to study how the Atg1/ULK1 complex integrates the signals that induce autophagy in accordance with fungus to mammalian autophagy regulation pathways. This framework lays the foundation for studying the deeper molecular mechanisms of the Atg1 complex in pathogenic fungi.

## Introduction

Autophagy is a conserved intracellular degradation pathway and a self-protective mechanism that maintains normal life activities from yeast to mammals (Melendez et al., 2003; Nakagawa et al., 2004; Hara et al., 2006; Komatsu et al., 2006; Mizushima et al., 2008; Mizushima and Komatsu, 2011; Farre and Subramani, 2016; Dikic and Elazar, 2018; Levine and Kroemer, 2019). It is also the main pathway for the production of new building blocks and energy for cellular homeostasis *via* the recycling of cytosolic entities. There are three primary classes of autophagy: macroautophagy, microautophagy, and chaperone-mediated autophagy (CMA). Macroautophagy has been extensively studied. This form of autophagy is initiated in the pre-autophagosomal structure (PAS), which is localized in the vicinity of the vacuole in *Saccharomyces cerevisiae* and *Magnaporthe oryzae* (Suzuki et al., 2001). The initiation of autophagy in mammals is located in the endoplasmic reticulum (ER) subdomain, which is known as the omegasome (Axe et al., 2008; Schutter et al., 2020). The phagophore elongates into a cup-shaped structure from the PAS or omegasome and eventually matures to form autophagosomes. The outer membrane of the autophagosome with the lysosome (vacuole in yeast or plants) forms the autolysosome, where the inner membrane and the captured materials are degraded (Liu et al., 2012).

A series of autophagy-related (Atg) genes were studied in yeast (Klionsky et al., 2003). More than 40 conserved Atg proteins that are functionally important for autophagy have been identified using genetic screening in *S. cerevisiae* and other fungal species (Sun et al., 2013). Among these autophagy-related proteins, more than 20 are necessary for autophagosome formation. These proteins are classified into 6 functional groups: the Atg1 kinase complex, the transmembrane protein Atg9 (Sawa-Makarska et al., 2020), an autophagy-specific phosphatidylinositol 3-kinase (PI3K) complex, the Atg2-Atg18 complex (Mizushima et al., 2011), and the Atg8 and Atg12 conjugation systems (Ichimura et al., 2000; Nakatogawa et al., 2007; Kaufmann et al., 2014; Wen and Klionsky, 2016). Most of these core Atg proteins possess mammalian homologs, which suggests that these proteins are highly conserved in eukaryotes.

These Atg proteins are recruited to PAS sites in a hierarchical manner (Fujioka et al., 2020). Among the six functional groups, Atg1 kinase complex is the most upstream (Suzuki et al., 2007) and a key initiator of autophagy. Upon starvation, Atg1 is recruited to PAS sites to initiate autophagy. This complex contains 5 proteins in *S. cerevisiae*, including Atg1, Atg13, Atg17, Atg29, and Atg31. Assembly of the Atg1 complex contributes to the kinase activity of Atg1, which is essential for the regulation of autophagy (Kamada et al., 2000). The ULK1/2 complex is the mammalian homolog of the Atg1 complex in yeast, which consists of 3 core subunits ULK1/2, RB1CC1/FIP200, Atg13, and a unique Atg101 subunit (Nanji et al., 2017). TOR (the target of rapamycin) and AMPK (AMP-activated protein kinase) regulate Atg1/ULK1 kinase activity *via* a network of phosphorylation events. The Atg1/ULK1 complex is associated with downstream regulators involved in various steps of autophagy.

Recent studies have found that the Atg1/ULK1 complex plays multiple roles in autophagosome formation and autophagy induction. This review discusses how the Atg1/ULK1 complex responds to the upstream signaling of TOR and AMPK and how the Atg1/ULK1 complex regulates autophagy process including autophagosome membrane expansion, autophagosome maturation, and autophagosome-vacuole fusion. Furthermore, we summarize the essential functions of the Atg1/ULK1 complex in different fungi. These new findings may help researchers understand the regulatory mechanism of the Atg1/ULK1 complex and provide a theoretical framework for further research on the molecular regulatory mechanism of the Atg1/ULK1 complex in pathogenic fungi.

## Atg1/ULK1 complex formation and structure

A comprehensive understanding of the structure and composition of the Atg1/ULK1 complex will be helpful to understand the molecular regulatory mechanism of autophagy initiation. Here, we summarize the components of the Atg1/ULK1 complex, the assembly of the Atg1/ULK1 complex, and the similarities and differences of the Atg1/ULK1 complex in fungi and mammals.

### The components of the Atg1 complex in yeast

**Figure 1:**
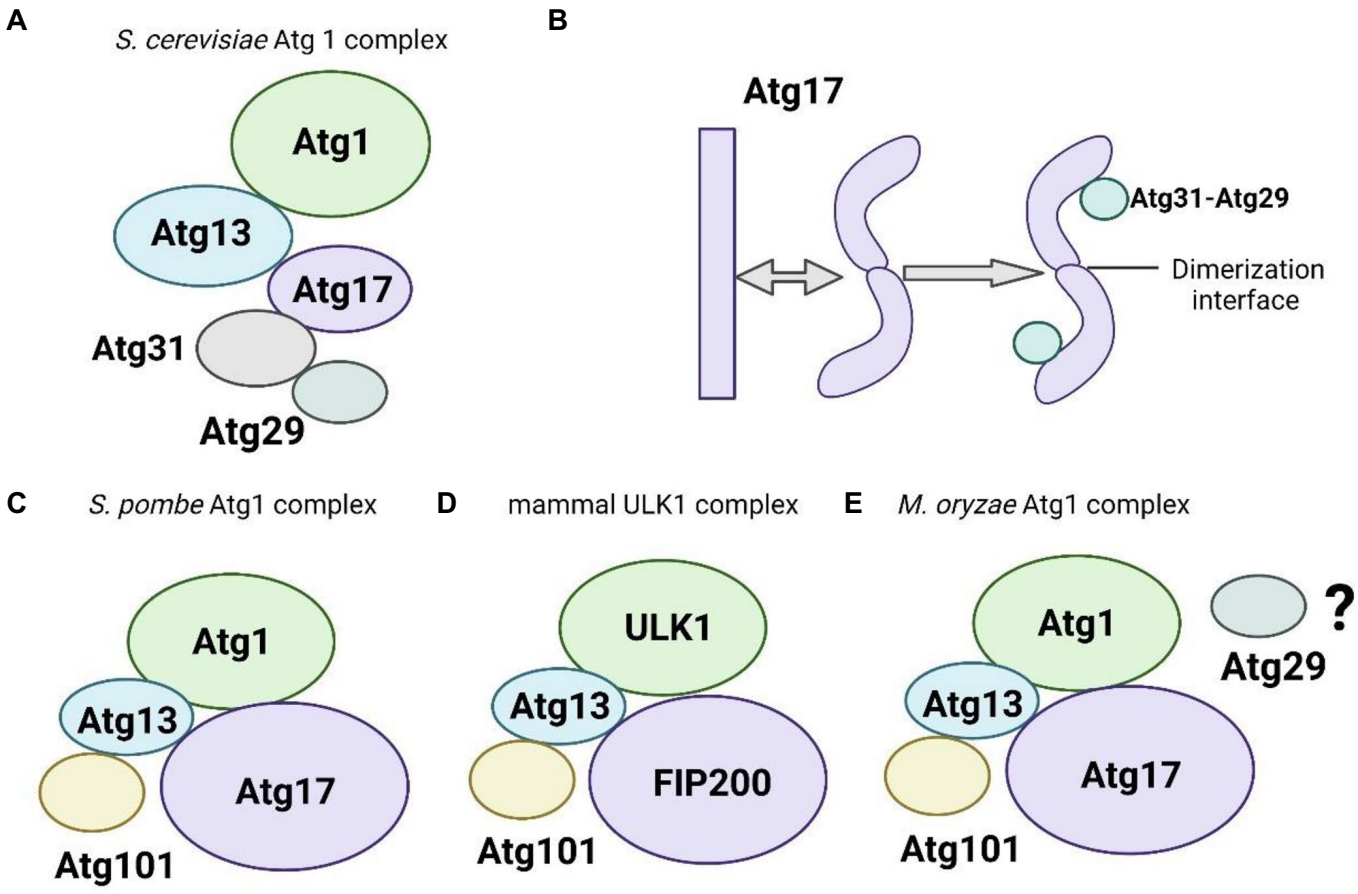
Schematic of the components of the Atg1/ULK1 complex. (**A)** The budding yeast *Saccharomyces cerevisiae* Atg1 complex components. **(B)** Components of the *S. cerevisiae* Atg17-Atg31-Atg29 complex with the depicted two smaller components Atg29 and Atg31 stabilizing the unique curvature of Atg17. **(C)** The fission yeast Atg1 complex components **(D)**. Mammalian ULK1 complex components. **(E)** The *M. oryzae* Atg1 complex components.

Recent biochemical and structural studies have emphasized the overall architecture of the *S. cerevisiae* Atg1 complex, which is comprised the Atg1 protein kinase, the regulatory protein Atg13, and dimerization of the Atg17-Atg31-Atg29 complex (Figure 1A). Atg17 is crescent-shaped with a 10-nm radius of curvature, which generates a central rod-like scaffold and mediates dimerization (Chew et al., 2013). The Atg17 and small Atg9-containing vesicles, which are precursors of the phagophore, exhibit resemble high curvature (Ragusa et al., 2012). The evidence suggests that the scaffolding subcomplex Atg17-Atg31-Atg29 targets the Atg1 kinase complex to the PAS by specifically recognizing the membrane protein Atg9. And the two regulatory subunits Atg31 and Atg29 inhibit this interaction (Rao et al., 2016). When *ATG29* is deleted in budding yeast *S. cerevisiae*, autophagy induction is severely blocked (Kawamata et al., 2005). *ATG31* and *ATG29* play key roles in stabilizing the unique curvature of Atg17. When *ATG31* and *ATG29* are deleted, Atg17 adopts multiple conformations instead of a single conformation (Figure 1B; Chew et al., 2013).

X-ray crystallography technology analysis found the interactions of yeast Atg13 with Atg1 and Atg17 (Fujioka et al., 2014). The microtubule interacting and transport (MIT) domains of Atg1 bind to 2 cognate MIT-interacting motifs (MIM) of Atg13 *via* hydrophobic interactions, and Atg17 interacts with Atg13 using a short region. These interactions allow Atg13 to bridge the Atg17 central scaffold to Atg1 (Fujioka et al., 2014). The C-terminal domain (CTD) of Atg13 and Atg1[CTD] binds to the tip regions of Atg17-Atg31-Atg29. The inter-subunit interactions within the Atg1 complex were further identified using chemical crosslinking coupled with mass spectrometry analysis (Chew et al., 2015). The Atg1 and Atg13 central domains together form a stable complex with ~100 nM affinity. Upon starvation and target of rapamycin complex 1 (TORC1) inactivation, Atg1 and Atg13 assemble with the Atg17-Atg31-Atg29 scaffold with ~10 μM affinity *via* Atg13, which initiates autophagosome formation (Stjepanovic et al., 2014).

Whether different subunit compositions impact the overall architecture of the *S. cerevisiae* Atg1 complex and the mammalian ULK1/2 complex is not clear. Recent studies in fission yeast *S. pombe* have established a simplified model to elucidate this issue. Unlike budding yeast *S. cerevisiae*, the subunit composition in the fission yeast *S. pombe* Atg1 complex resembles the human ULK1 complex containing Atg101 (Figure 1D). Atg101 does not bind to Atg1, probably because the putative scaffold subunit Atg17 adopted a rod-shaped structure with no discernible curvature in negative stain electron microscope studies in *S. pombe*. Atg101 binds the closed Hop1, Rev7, and Mad2 (HORMA) domain of Atg13. And Atg101 also interacts with the CTD of Atg1, which suggests that this unique subunit has functions beyond stabilizing Atg13 HORMA.

### Components of the ULK1/2 complex in mammals

The ULK1/2 complex, which is the mammalian ortholog of the yeast Atg1 complex, consists of 4 core subunits, ULK1/2, Atg13, FIP200/Atg17, and the metazoan-specific subunit Atg101 (Figure 1C). FIP200 (also known as RB1CC1) is a large predicted coiled-coil protein that functionally resembles the two scaffold proteins Atg17 and Atg11 in yeast (Mizushima, 2010; Lin and Hurley, 2016). And the N-terminal domain (NTD) of the FIP200 forms a C-type scaffold, providing a hub for the assembly of the ULK1 complex (Shi et al., 2020b). Besides, FIP200 organizes autophagic mechanisms at p62-ubiquitin condensates beyond to ULK1 kinase activation (Turco et al., 2020). And FIP200 coiled-coil and autophagic adapter NDP52 allosterically activate ULK1 complex membrane recruitment (Shi et al., 2020a). Atg13 contains a long intrinsically disordered region (IDR) following the HORMA domain, and the C-terminal part of its IDR binds to the C-terminal two tandem MIT (tMIT) domains of ULK1 (Fujioka et al., 2014). Atg101 and Atg13 exhibited an open HORMA domain fold conformation that can heterodimerize with each other (Hegedus et al., 2014; Michel et al., 2015; Qi et al., 2015; Suzuki H. et al., 2015). This heterodimeric subcomplex enhances the stability of both proteins and the recruitment of downstream Atg proteins (Noda and Mizushima, 2016). Obviously, Atg29 and Atg31 are essential to stabilize the S-shaped architecture of Atg17 in *S. cerevisiae* (Kawamata et al., 2005; Kabeya et al., 2009; Chew et al., 2013). Atg101 is evolutionarily conserved in eukaryotes. However, the sequence of Atg101 is not similar to Atg29 or Atg31 (Noda and Mizushima, 2016).

### Components of the Atg1 complex in the plant pathogenic fungus *Magnaporthe oryzae*

**Figure 2:**
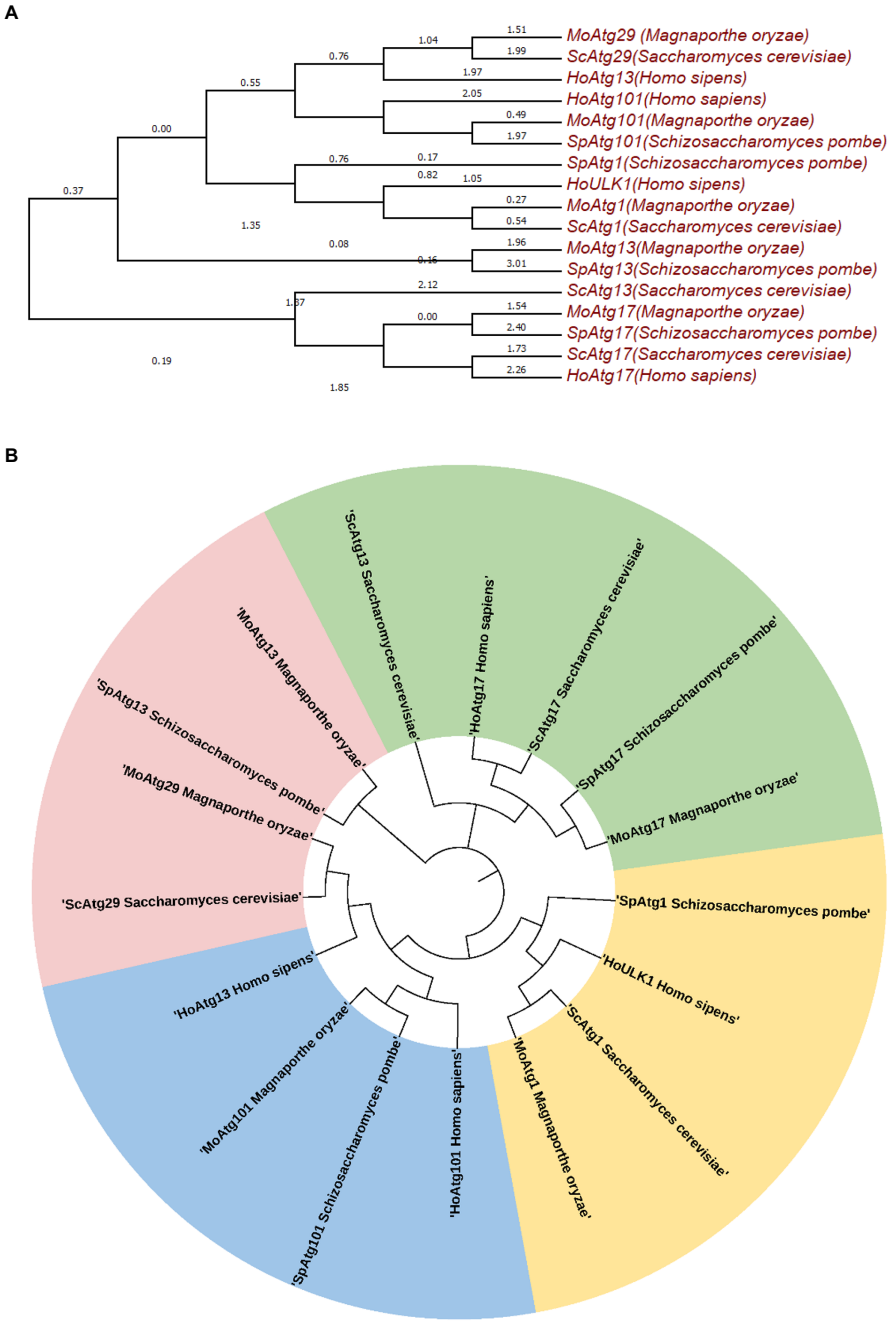
Molecular phylogenetic analysis by the maximum likelihood method **(A,B)**. The evolutionary history was inferred using the maximum likelihood method based on the JTT matrix-based model. The tree with the highest log likelihood (−7283.2388) is shown. Initial tree(s) for the heuristic search are obtained automatically by applying the neighbor-joining and BioNJ algorithms to a matrix of pairwise distances estimated using a JTT model then selecting the topology with a superior log likelihood value. The analysis involved 17 amino acid sequences. All positions containing gaps and missing data were eliminated. There is a total of 160 positions in the final dataset. Evolutionary analyses were performed in MEGA7.

The Atg1 complex in *M. oryzae*, contains five core subunits, Atg1, Atg13, Atg17, Atg29, and Atg101 (Figure 1E; data not published). We constructed an evolutionary tree based on the JTT matrix-based model using the maximum likelihood method. The results showed that *MoATG29*, *MoATG1,* and *MoATG13* of *M. oryzae* were in the same branch with *S. cerevisiae*. *ScATG29*, *ScATG1,* and *ScATG13*, with 36.84%, 25.43%, and 26.60% homology, respectively. *MoATG17* in *M. oryzae* and *SpATG17* in *S. pombe* were evolutionarily conserved and in the same branch (Figures 2A,B). Previous studies in our laboratory have identified the serine/threonine kinase MoAtg1, which is screened in the Blast in GenBank from a differentially subtractive suppression library for genes that are highly expressed at the appressorium stage (Lu et al., 2005; Liu et al., 2007b). Subsequent studies in our laboratory found that MoAtg1, MoAtg13, and MoAtg17 interacted with each other using yeast two-hybrid methods, and the interaction between Atg101 and Atg13 was conserved in *M. oryzae* (data not published), *S. pombe,* and mammals. However, the composition of the interaction with MoAtg29 is not clear, and further studies will be performed.

### The structure of the Atg1/ULK1 complex among in yeast, mammals, and rice blast fungus

To better compare the structural differences of the Atg1 complex in different organisms, we need atomic-resolution structural data. However, structurally relevant information of most proteins in protein databases is limited. The newly developed protein prediction platform AlphaFold2, which is based on machine learning methods, has extended the coverage of protein structures with high accuracy.

**Figure 3:**
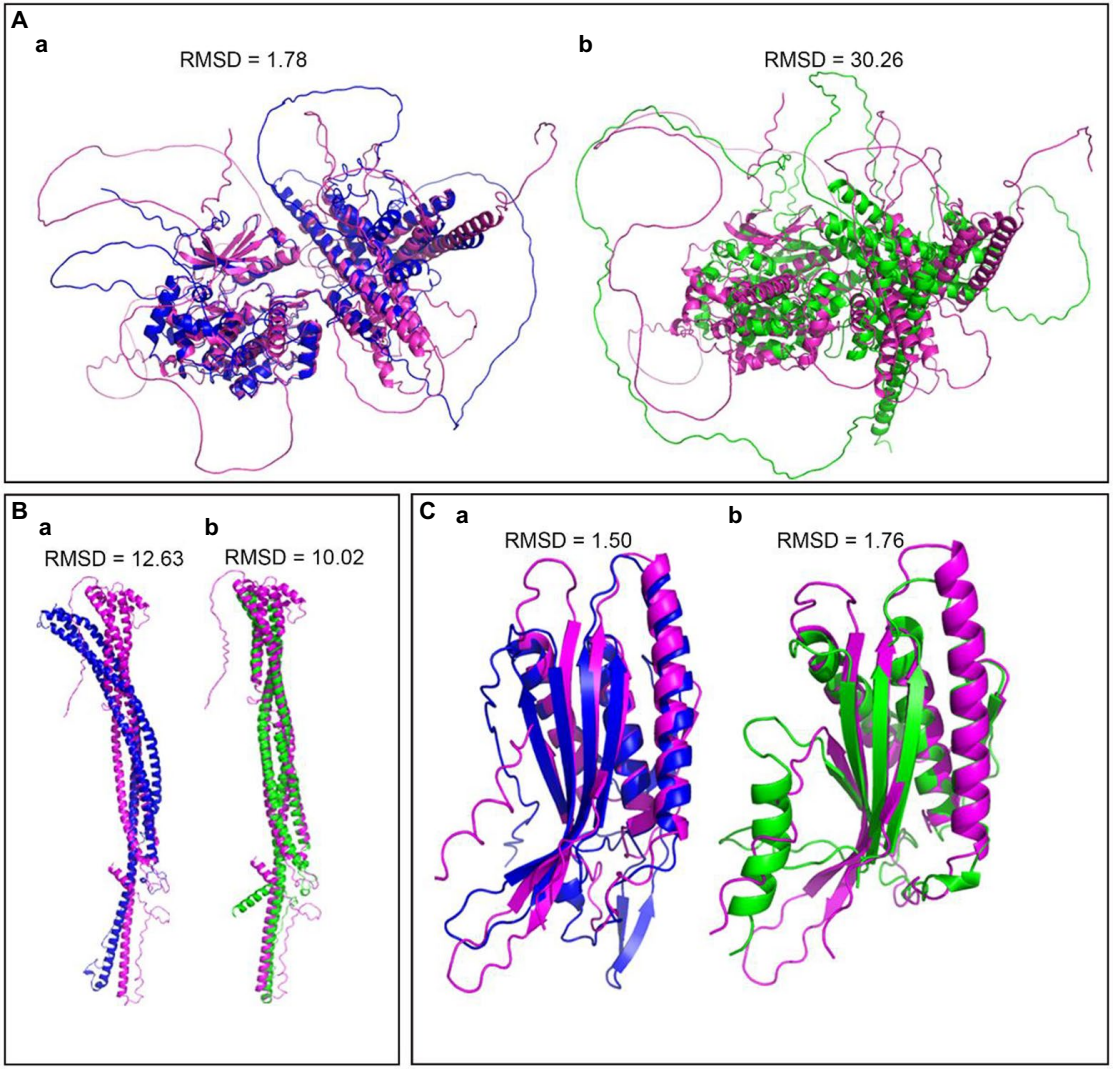
Structural differentiation of the Atg1 complex between rice blast fungus, yeast and mammals. **(A)** The predicted structure of Atg1 by Alphafold2, Pink-MoAtg1, Bule-ScAtg1, green-SpAtg1. **(B)** The predicted structures of Atg17, Pink-MoAtg17, Bule-ScAtg17, green-SpAtg17. **(C)** The predicted structures of Atg101, Pink-MoAtg101, Bule-HoAtg101, and green-SpAtg101. RMSD (Root Mean Square Deviation) value is used to measure the similarity of two structures. A smaller RMSD value indicates a higher degree of similarity.

To compare the structures of the components in the Atg1/ULK1 complex, we submitted amino acid sequences of Atg1 and Atg17 among *M. oryzae*, *S. cerevisiae,* and *S. pombe* to the AlphaFold2 database, and the structures were well simulated. The results indicated that Atg1 protein was conserved in rice blast fungus and yeast (Figures 3A,B). We also submitted MoAtg101, HsAtg101, and SpAtg101 amino acid sequences to the AlphaFold2 database, and the structures were well simulated. These results indicated that the Atg101 protein was conserved in rice blast fungus, fission yeast, and mammals (Figure 3C).

## The upstream regulators of the Atg1/ULK1 complex

**Figure 4:**
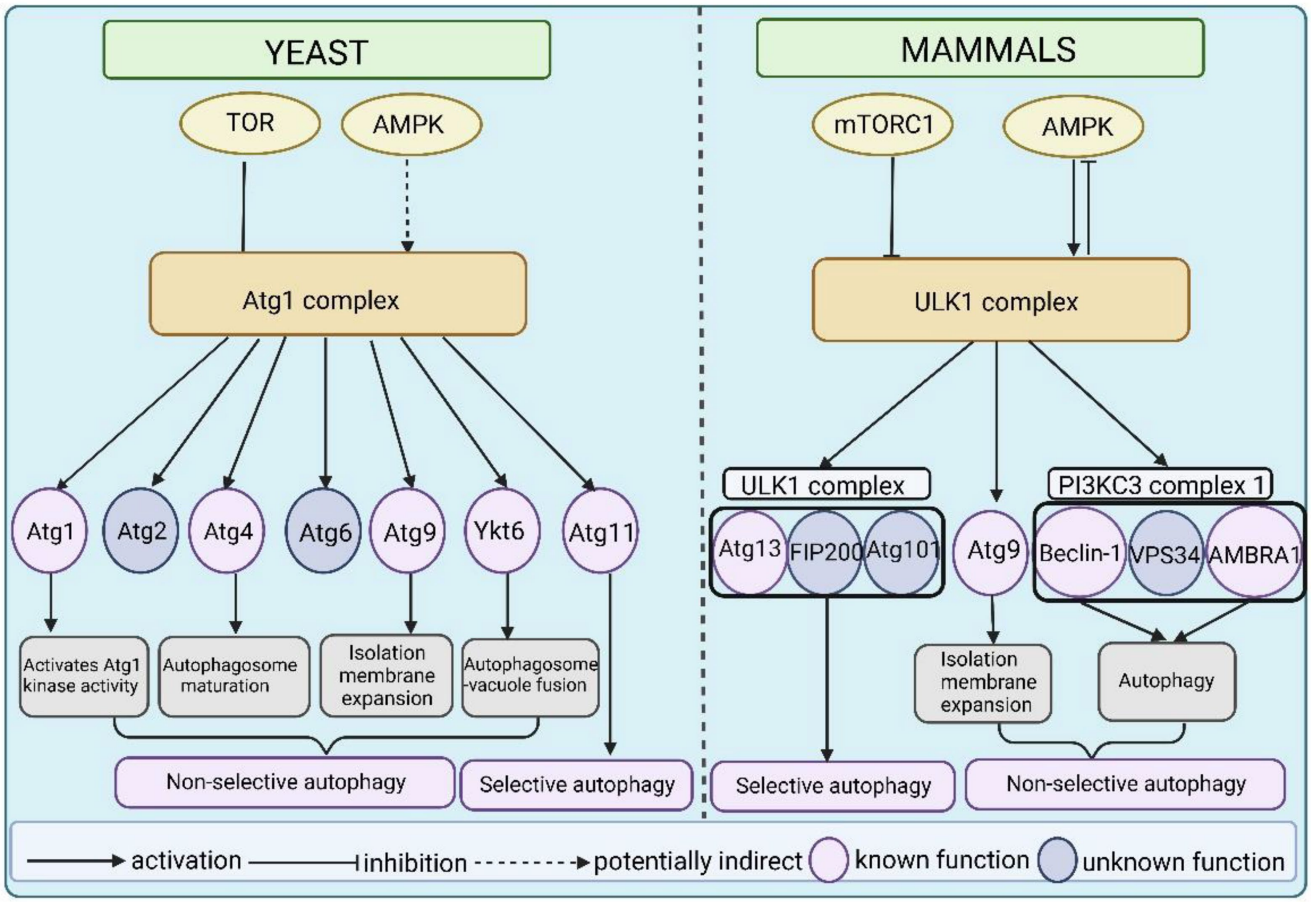
A model of the Atg1/ULK1 complex as an important hub linking upstream signals and downstream regulators in yeast and mammals. The activity of Atg1 and ULK1 is directly or indirectly regulated by TOR and AMPK. In yeast, Atg1 autophosphorylation is essential for activating Atg1 kinase activity. Atg1 phosphorylates Atg4 to promote autophagosome maturation. Atg1 phosphorylates Atg9 to promote isolation membrane expansion. Atg1 phosphorylates Ykt6 to control autophagosome–vacuole fusion. And Atg1 phosphorylates Atg11 and is essential for selective autophagy. Atg1 also phosphorylates Atg2 and Atg6, but the function of these phosphorylation events is not clear. In mammals, ULK1 phosphorylates its complex binding components Atg13, FIP200, and Atg101. Among these components, phosphorylation of Atg13 by ULK1 plays an important role in mitophagy, but the function of phosphorylation of the other two remains unclear. Similar to yeast, ULK1 phosphorylates Atg9 to regulate isolation membrane expansion. The phosphorylation of beclin-1 and AMBRA1 is required for autophagy induction but the function of Vps34 phosphorylation is not clear.

The Atg1/ULK1 complex is critical in autophagy induction, and it is a major hub in the regulatory pathway that receives upstream autophagy initiation signals and transmits them to downstream regulators (Figure 4). Atg1/ULK1 and their complex components are phosphorylated by several signaling kinases, including the TOR kinase complex, AMPK, autophagy beclin 1 regulator 1 (AMBRA1), and PKA (protein kinase A; Noda and Ohsumi, 1998; Budovskaya et al., 2004; Schmelzle et al., 2004; Meley et al., 2006; Stephan et al., 2009). TOR and AMPK-activated kinases are the main kinases that sense external environmental nutrient signals and transmit them to the Atg1/ULK1 kinase complex. This review will focus on the regulation of the Atg1/ULK1 kinase complex by TOR and AMPK.

### Regulation by TOR in *Saccharomyces cerevisiae*

TOR was first described in yeast in 1991 as a target protein of the immunosuppressive compound rapamycin (Heitman et al., 1991). Subsequent studies revealed the essential roles of TOR kinase in autophagy of yeast and higher eukaryotes, including terminating autophagy under nutrient-rich conditions and enhancing autophagy during starvation or rapamycin treatment (Wullschleger et al., 2006; Yu et al., 2010; Yang et al., 2013; Nazio and Cecconi, 2017; Saxton and Sabatini, 2017). The Atg1 complex assembles and functions in a nutrient-dependent manner in *S. cerevisiae*. Under abundant nutrient conditions, TOR directly phosphorylates Atg13 at multiple sites, and eight potential TOR phosphorylation residues were identified in Atg13 using sequence similarity and mass spectrometry approaches (Kamada et al., 2010). Among these residues, the Atg13 Ser496 residue had a striking effect on autophagy induction, which was revealed in the crystal structure of the Atg13 regions interacting with Atg1 and Atg17 (Fujioka et al., 2014). The hyperphosphorylated form of Atg13 has a very low affinity for Atg1 and inhibits autophagy (Kawamata et al., 2008). Under nutrient starvation, the activity of TOR is inhibited, which suppresses Atg13 phosphorylation. The Atg13 dephosphorylation strengthens the interaction between Atg1 and Atg17, which is consistent with the upregulation of Atg1 kinase activity. And this interaction contributes to the assembly of Atg1 complex at the PAS, which then activates autophagy process (Kawamata et al., 2008).

Atg1 is a conserved serine/threonine kinase and a direct substrate of TOR (Matsuura et al., 1997). Some studies showed that TOR activity regulated five phosphorylation sites of Atg1 (Matsuura et al., 1997; Budovskaya et al., 2005; Kijanska et al., 2010; Yeh et al., 2010, 2011). The phosphorylation of four in these residues is upregulated in rapamycin treatment conditions and evolutionarily conserved. Two phosphorylation residues Thr226 and Ser230, which are located in the kinase activation loop, are essential for Atg1 kinase activity and function in autophagy (Kijanska et al., 2010). Thr226 phosphorylation requires Atg1 autophosphorylation and two core complex components Atg13 and Atg17 (Yeh et al., 2011). Overall, these studies have shown that five phosphorylation sites within Atg1 regulated by TOR play a critical role Atg1 kinase activity and function in autophagy.

### Regulation by TOR in *Magnaporthe oryzae*

Similar to *S. cerevisiae*, the regulatory protein TOR directly phosphorylates Atg13 in *M. oryzae* under normal growth conditions, which prevents its interaction with Atg1. However, when pathogenic fungi are stressed by the external environment, such as nutrient deficiency, injury, hypoxia, and reactive oxygen species (ROS) accumulation or treated with rapamycin, the activity of TOR decreases and then Atg13 undergoes dephosphorylation. Dephosphorylated Atg13 binds to Atg17, which promotes the formation of the Atg1-Atg13-Atg17 complex. The localization of this complex at the PAS marks the initiation of autophagy (Liu et al., 2007a).

An increasing number of studies have shown that TOR is essential for multiple life activities of *M. oryzae*. The precise regulation of TOR activity plays an indispensable role in the process of spore germination, appressorium formation, and infection (Marroquin-Guzman and Wilson, 2015). During the appressorium formation stage, TOR activity is reduced due to the lack of external nutrients, and then cell division is blocked. The appressorium turgor increased due to the accumulation of glycerol. An upstream negative regulator of TOR, MoAsd4, inhibits TOR activity by regulating amino acid metabolism, which further promotes appressorium formation (Marroquin-Guzman and Wilson, 2015). The AMPK β subunit protein-like protein MoAbl1 acts as a negative upstream regulator of TOR and inhibits TOR activity in the presence of glucose deficiency. In contrast, the glucose-6-phosphate receptor protein trehalose-6-phosphate (T6P) synthase protein MoTps1 activates TOR by regulating the NADPH-dependent glucose signaling pathway to facilitate the nuclei of appressorium into the infected mycelium and initiate cell division (Marroquin-Guzman et al., 2017). Recent studies have identified several downstream regulatory elements of TOR, including a major component of the Atg1 kinase complex MoAtg13, the epigenetic factor MoSnt2, MoTap42 (type 2A associated protein of 42 kDa), a novel vacuolar protein MoImp1, the sterol binding protein MoVast1 (a VASt domain-containing protein), and MoOpy2 (overproduction-induced pheromone-resistant protein 2; Liu et al., 2007a; He et al., 2018; Qian et al., 2018; Sun et al., 2018; Zhu et al., 2021; Cai et al., 2022). These studies have suggested that TOR also plays key roles in cell autophagy and cell integrity.

### Regulation by TOR in mammals

Consistent with yeast and *M. oryzae*, mTORC1, the TOR homolog in higher eukaryotes, is a major negative regulator of autophagy (Wullschleger et al., 2006; Settembre et al., 2011) and regulates the kinase activity of ULK1. Under starvation or rapamycin conditions, mTOR is inhibited, which induces autophagy and causes the dephosphorylation of ULK1, ULK2, and Atg13 (Jung et al., 2009). Mammalian Atg13 interacts with ULK1 and ULK2 which activate ULK kinase, and facilitate the phosphorylation of FIP200 *via* ULK1. Atg13 and FIP200 can enhance ULK1 kinase activity under maximal stimulation (Jung et al., 2009). In contrast to yeast TOR, mTOR in mammals phosphorylates Atg13 and ULK1 under nutrient-rich conditions, which inhibits ULK1 functions (Hosokawa et al., 2009; Jung et al., 2009). Consistent with this effect, the ULK1 complex strongly associates with mTORC1 in nutrient-rich conditions (Hosokawa et al., 2009). Two phosphorylation sites were identified in ULK1 as mTORC1 substrates using mass spectrometry (Alers et al., 2012; Mack et al., 2012).

Under nutrient-rich conditions, activated mTORC1 phosphorylates the Ser758 site in ULK1, which prevents ULK1 from interacting with AMPK (Kim et al., 2011; Shang et al., 2011). The Ser758 site of ULK1 is only phosphorylated by mTORC1, while the Ser638 site of ULK1 is phosphorylated by mTORC1 and AMPK (Shang et al., 2011). This evidence suggests that mTOR directly regulates the ULK-Atg13-FIP200 complex in response to autophagy induction. The interaction between Atg1 and Atg13 is regulated by rapamycin and nutrient deficiency in *S. cerevisiae* and *M. oryzae*. Whereas, nutrient conditions do not alter the interaction between ULK1 and mammalian Atg13 (Hosokawa et al., 2009; Jung et al., 2009). One possible explanation is that mTOR-mediated phosphorylation does not lead to extreme conformational changes in ULK1 and Atg13. However, recent studies have shown that mTORC1 directly regulates ULK1 kinase activity by phosphorylating unidentified ULK1 residues and indirectly *via* AMBRA1, which increased ULK1 kinase activity and stability by AMBRA1 dephosphorylation and activation of ULK1 ubiquitylation (Nazio et al., 2013). ULK1 facilitates AMBRA1 *via* phosphorylation. This mutual regulation homeostasis mechanism may be essential to human diseases due to impaired autophagy. Additionally, mTORC1 controls autophagy separately from the Atg1/ULK1 complex pathway from yeast to mammals. For example, the regulated expression of Atg14 contributes to autophagy in *S. cerevisiae* (Chan et al., 2001), and mTORC1 regulates autophagy *via* an altered transcription pattern in yeast and higher eukaryotes (Schmelzle and Hall, 2000).

### The upstream regulators AMPK/Snf1

AMPK is a critical cellular energy sensor and regulates metabolism under energy-stress conditions. AMPK is essential to the regulation of autophagy mediated by the Atg1/ULK1 complex from yeast to mammals (Knighton et al., 1991; Wang et al., 2001; Meley et al., 2006; Hoyer-Hansen et al., 2007). The homolog of AMPK in the yeast *S. cerevisiae*, *SNF1*, may regulate autophagy *via* Atg1 and Atg13 according to a genetic screen (Wang et al., 2001). AMPK regulates autophagy by molecular manipulations *via* different pathways in higher eukaryotes. TSC2 tumor suppressor is phosphorylated by AMPK on Thr1227 and Ser1345 (Inoki et al., 2003) and mTOR binding partner raptor is phosphorylated on Ser722 and Ser792 (Gwinn et al., 2008). These phosphorylation modifications negatively regulate mTORC1 activity to activate autophagy. However, AMPK directly phosphorylates ULK1 on Ser467, Ser555, Thr574, and Ser637. All of these phosphorylation events are essential for autophagy and mitophagy (Gwinn et al., 2008). AMPK-dependent phosphorylation contributes to ULK1 activation in an mTOR-indepedent pathway, which impacts the recruitment of downstream factors, such as Atg9 (Bach et al., 2011; Egan et al., 2011; Mack et al., 2012). ULK1 also phosphorylates three subunits of AMPK to regulate its activity. The negative feedback mechanism between AMPK and ULK1 better controls the autophagy level (Loffler et al., 2011). Notably, some studies suggested that AMPK interacted with ULK1 in a nutrient-dependent manner (Loffler et al., 2011; Shang et al., 2011; Mack et al., 2012). TORC1 inhibits the interaction between ULK1 and AMPK, and ULK1 phosphorylation on Ser757 by mTORC1 promotes this interaction (Kim et al., 2011; Shang et al., 2011), but the specific regulatory mechanism of this interaction is not known.

## Atg1/ULK1 substrates

Recently, the identification of Atg1 substrates has been gradually clear. The yeast *S. cerevisiae* is a typical model organism for autophagy research. Recent studies have identified Atg1 substrates in yeast, including Atg1, Atg2, Atg4, and Atg9, SNARE (soluble N-ethylmaleimide sensitive factor attachment protein receptor) protein Ykt6, and Atg11 (Kijanska et al., 2010; Yeh et al., 2010; Liu et al., 2012; Papinski et al., 2014; Sanchez-Wandelmer et al., 2017; Barz et al., 2020; Gao et al., 2020). Atg1 directly phosphorylates Atg2 at Ser249 and Ser1086 in cell culture (SILAC) quantification; however, mutations of both phosphorylation residues have no impact on autophagy under nutrient starvation conditions (Papinski et al., 2014), which suggests a redundancy of both phosphorylation sites. Therefore, we primarily concentrated on the phosphorylation of the other five proteins mediated by Atg1. Notably, Atg1 is involved in multiple autophagic processes in non-selective autophagy, including isolation membrane expansion, autophagosome maturation, autophagosome-vacuole fusion, and selective autophagy by phosphorylating Atg4, Atg9, Ykt6, and Atg11. The ULK1 kinase is closely associated with autophagosome formation and vesicular transport in mammals *via* the phosphorylation of many downstream substrates (Papinski and Kraft, 2016). Different from other Ser/Thr kinases, the special feature of ULK1 is a preference for hydrophobic sites around the phosphorylation residues (Papinski et al., 2014; Egan et al., 2015). Recent studies have shown that ULK1 substrates contain members of the ULK1 complex (Atg13, FIP200, and Atg101), Atg9, and PI3KC3-C1 subunits (beclin-1, Vps34/PIK3C3, and AMBRA1; Fimia et al., 2007; Chan et al., 2009; Di Bartolomeo et al., 2010; Russell et al., 2013; Wirth et al., 2013; Hurley and Schulman, 2014; Egan et al., 2015; Karanasios et al., 2016; Papinski and Kraft, 2016). Therefore, we will focus on the phosphorylation of these substrates mediated by ULK1.

### Atg1 substrates in yeast

Previous studies have reported that Atg1 has two phosphorylated residues Thr226 and Ser230 using mass spectrometry methods. Both phosphorylation sites are located in the activation loop of the amino-terminal kinase domain of Atg1 and are conserved from yeast to mammals (Yeh et al., 2010). Mutation of either site leads to Atg1 kinase inactivation and abolishes its role in autophagy. Phosphorylation of Thr226 and Ser230 directly activates Atg1 kinase without impacting Atg1 complex formation or localization at the PAS. Phosphorylation of Thr226 requires not only the presence of Atg13 and Atg17, but also the autophosphorylation activity of Atg1 (Yeh et al., 2010). Notably, a small fraction of Atg1 formed dimers or oligomers and this dimerization triggers Atg1 kinase activation *via* trans-autophosphorylation (Kijanska et al., 2010; Yeh et al., 2010). However, the upstream regulators that induce Atg1 dimerization are not clear. Furthermore, Atg1-mediated phosphorylation of Atg13 triggers the dissociation of the Atg1 complex (Schreiber et al., 2021) and PAS-localized protein phosphatases dephosphorylate Atg13 (Memisoglu et al., 2019), thus potentially promoting rapid recombination of the Atg1-based complex.

Recent studies have proposed a novel model in which Atg1 transiently inhibits the deconjugating activity of Atg4 *via* the phosphorylation of Ser307 at the PAS, which prevented Atg4 from interacting with Atg8-phosphatidylethanolamine (PE). This novel regulatory mechanism protects the synthesis and maintenance of the Atg8-PE pool, which is essential for autophagosome biogenesis (Sanchez-Wandelmer et al., 2017). When the formation of an autophagosome is completed, inactivated Atg1 allows Atg4 to process the Atg8-PE on autophagosomal membranes and recycle Atg8, which is involved in a new round of autophagy (Nakatogawa et al., 2007; Kaufmann et al., 2014). Consistent with this pathway, ULK1 inhibits Atg4B (the mammalian homolog of Atg4) *via* phosphorylation at Ser316 (Pengo et al., 2017). However, mutation of the corresponding Ser354 in yeast showed no impairment in autophagy. Overall, these results indicate a conserved regulatory mechanism that Atg1 homologs inhibit Atg4-deconjugating activity *via* phosphorylation. ULK1 also inhibits human Atg4B but uses different serine residues (Pengo et al., 2017). Notably, further studies are required to identify the upstream regulators that control Atg1 kinase activity in a timely manner in this novel model.

Atg9 is present in Atg9 vesicles, which are cytoplasmic mobile vesicles with 30–60 nm diameter, and it is derived from the Golgi apparatus (Puri et al., 2013). It is predicted to have six membrane-spanning domains. Under nutrient starvation conditions, approximately three Atg9 vesicles are recruited to the PAS and integrated into the outer autophagosomal membrane. After completion of autophagosome formation, Atg9 vesicles are recycled to the cytoplasm and participate in a new round of autophagosome formation (Yamamoto et al., 2012). The transmembrane protein Atg9 is a direct target of Atg1 in consensus peptide arrays, and Atg1 phosphorylates Atg9 on several residues *in vitro* and *in vivo*. Phosphorylated Atg9 is essential for the recruitment of Atg8 and Atg18 to the PAS and contributes to the expansion of the isolation membrane, which is required for autophagy (Papinski et al., 2014). However, Atg9 is still recruited to the PAS under *ATG1* deletion conditions. Mutation of *ATG1* phosphorylation sites does not alter Atg9 localization (Reggiori et al., 2004). When Atg9 is recruited to the PAS, the Atg13 HORMA domain directly associates with the amino-terminal cytosolic stretch of Atg9 (Suzuki S. W. et al., 2015). Atg17 also directly interacts with Atg9 (Rao et al., 2016). Notably, Atg9 reaches the PAS but cannot return to the cytosolic pools without the Atg1 complex (Reggiori et al., 2004). Taken together, Atg9 binds to the PAS and returns to the cytoplasm independent of Atg1 kinase activity, but the Atg1 complex is essential for this process.

SNARE proteins are important for the fusion of mature autophagosomes with vacuoles (Moreau et al., 2011; Nair et al., 2011). Generally, SNARE-mediated fusion is achieved *via* the formation of one R-SNARE and three Q-SNARE proteins, which are facilitated by Rab GTPases and tethering complexes. R-SNARE Ykt6 forms a SNARE bundle with Q-Snares Vam3, Vti1, and Vam7 on vacuoles in yeast to act on autophagosomes (Bas et al., 2018; Gao et al., 2018). Ykt6 is involved in many membrane-transport events such as cis-Golgi retrograde transport and homotypic vacuolar fusion (Sogaard et al., 1994; McNew et al., 1997; Tochio et al., 2001). Barz et al. recently have found that Atg1 is responsible for phosphorylation of Ser182 and Ser183 of Ykt6 during autophagosome-vacuole fusion, which prevents the assembly of Ykt6 to form a functional SNARE bundle (Barz et al., 2020). The bundle is inactivated because the interaction between the vesicular SNAREs Vam3 and Vti1 is disturbed, which prevents immature autophagosomes from fusing with vacuoles (Barz et al., 2020). Gao et al. also have found that Ykt6 is an important target of the kinase Atg1 and participates in the fusion of autophagosomes and vacuoles. Ykt6 is located at the PAS site before autophagosome formation (Gao et al., 2020). This study supports the finding of Barz et al., who have found that Atg1 kinase regulates the fusion of autophagosomes with vacuoles in a timely manner to avoid the degradation of immature autophagosomes by vacuoles (Barz et al., 2020). This finding is similar to the timely phosphorylation of Atg4 by Atg1 kinase to prevent Atg8 release when autophagosomes are immature, and it is responsible for autophagosome maturation (Sanchez-Wandelmer et al., 2017). Notably, Atg1 kinase regulates the process of autophagosome and vacuole fusion, and an unknown phosphatase dephosphorylates Ykt6 to enable autophagosome and vacuole fusion after autophagosome maturation, which is a direction worthy of further exploration.

Atg11 primarily plays the role as scaffold protein in selective autophagy. Atg11 interacts with substrate receptors for degradation to promote autophagosome formation. And it can also interact with other biomacromolecules/complexes to mediate the phagophore to PAS (Lu et al., 2009). A large number of coiled-coil domains determine the functions of Atg11. Atg11 has four coiled-coil domains including CC1, CC2, CC3, and CC4 (Lu et al., 2009). The CC4 structural domain of Atg11 is associated with selective autophagy receptors which assist Atg11 in interacting with other proteins (Liu et al., 2015, 2017; Zhu et al., 2018; Shi et al., 2019; Sun et al., 2022). Yao et al. recently have found that phosphorylated residues Ser949, Ser1057, and Ser1064 in the CC4 domain of Atg11 by Atg1 participate in the regulation of selective autophagy, including the cytoplasmic vacuolar targeting (Cvt) pathway, mitophagy, endoplasmic reticulum autophagy, and peroxisome autophagy in *S. cerevisiae* (Liu et al., 2012). Recent studies have reported that Atg11 is involved in the activity of Atg1 kinase in *S. pombe*, and Atg1 is activated by Atg11-mediated dimerization (Farre and Subramani, 2016). Further studies are needed to elucidate whether Atg1 kinase activity regulates the function of Atg11 in *S. pombe*. Taken together, the phosphorylation of Atg11 by Atg1 kinase is involved in selective autophagy. However, whether Atg11 has other post-translational modifications that also affect its activity or whether it is a substrate for other protein kinases is not clear and deserves further investigation.

### ULK1 substrates in mammals

ULK1 directly phosphorylates components of the ULK1 complex, including Atg13, FIP200, and Atg101 (Egan et al., 2015). As mentioned above, ULK1 closely interacts with Atg13, and several studies have suggested that Atg13 is a direct target of ULK1 (Chan et al., 2009; Hosokawa et al., 2009). Researchers have found that Atg13 contains multiple phosphorylation sites that conform to the ULK1 substrate consensus sites (Egan et al., 2015). Besides, three FIP200 phosphorylation sites at Ser943, Ser986, and Ser1323 have been identified in a ULK1-dependent manner in humans (Egan et al., 2015). However, few studies showed the precise functions of these phosphorylation sites. The Atg101 phosphorylation sites Ser11 and Ser203 in humans conform to the ULK1 consensus substrate motif. However, the functional consequences have not been assessed (Egan et al., 2015).

Consistent with yeast, the interaction between Atg9 and ULK1 is also present in mammalian cells (Karanasios et al., 2016). ULK1 directly phosphorylates the transmembrane protein Atg9 which is essential for its translocation of the autophagy membrane (Karanasios et al., 2016).

PI3KC3 complex 1 is crucial for autophagosome formation and recruitment of autophagy downstream regulators (Pattingre et al., 2005; Miller et al., 2010; Wang et al., 2012; Kim et al., 2013; Wei et al., 2013). The core components of PI3KC3 complex 1, including beclin-1, Vps34/PIK3C3, and AMBRA1, are phosphorylated in a ULK-dependent manner (Di Bartolomeo et al., 2010; Russell et al., 2013). Under nutrient-depleted or rapamycin conditions, the activated ULK1 phosphorylates beclin-1 at the Ser15 site, which is required for autophagy induction in mammals (Russell et al., 2013). Consistent with this finding, the interaction between Atg1 and Atg6 is highly conserved in yeast (Kamber et al., 2015). When autophagy is induced, activated ULK1 phosphorylates AMBRA1 at Ser465 and Ser635 sites *in vivo* and *vitro* (Egan et al., 2015). Unlike beclin1 and AMBRA1, which have multiple ULK1-dependent phosphorylation sites, the lipid kinase Vps34 only contains a single ULK1 phosphorylation site at Ser249 and is evolutionarily conserved in yeast. However, phosphorylation of Ser249 in Vps34 is not essential for autophagy induction, which is likely attributed to redundant function or an unknown specialized function (Egan et al., 2015).

## Functions of the Atg1 complex in fungi

Autophagy plays an extremely important role in hyphal development, conidia, reproduction, conidial germination, and nutrient metabolism during the growth and development of filamentous fungi. When autophagy is blocked, filamentous fungal cells will not be able to use nutrients to supply themselves in time, resulting in defects in growth, development, reproduction, and other aspects (Liu et al., 2017). With the gradual deepening of the study in filamentous fungal autophagy, it is found that autophagy not only has a regulatory effect on its growth and development, but also has an important impact on its pathogenicity. Filamentous fungi as pathogenic fungi, in the process of infecting the host plant, which usually form some special infection structures to break through the defense mechanisms such as the stratum corneum formed by plants during evolution (Liu et al., 2015, 2017). As a model fungus for filamentous fungi, *M. oryzae* has been studied more in terms of the impact of autophagy on its infection process and pathogenicity. In the infection process of the host plant by *M. oryzae*, it produces a specific infection structure, appressorium, where appressorium turgor pressure accumulates. And its nutrients are from the conidium, which provides material and energy for the infection process by degrading intracellular nutrients and organelles (Yin et al., 2019; Sun et al., 2022).

The key serine/threonine kinase Atg1, which is involved in autophagy initiation in *S. cerevisiae*, is important for a variety of cellular processes, such as autophagy, growth, differentiation, lipid metabolism, and virulence (Chan and Tooze, 2009). Previous studies have reported that Atg1 is involved in non-selective and selective autophagy pathways. Disruption of *ATG1* leads to a complete block of autophagy, pexophagy, and mitophagy (Yanagisawa et al., 2013). The Δ*atg1* mutant enhanced sensitivity to nitrogen starvation conditions compared to the wild type and reduced temporal and replicative lifespan and the loss of sporulation capacity in *S. cerevisiae* (Kamada et al., 2000; Pinan-Lucarre et al., 2003). Homologs of Atg1 were also confirmed in other fungi. However, the proteins encoded by the *ATG1*, *ATG13*, and *ATG17* genes in different fungi also have different functions. Here, we summarized the functions of the Atg1 complex in different fungi, including plant, insect, and human pathogenic fungi (Table 1).

**Table 1:** Atg1 complex related to pathogenicity in pathogenic fungi.

Gene	Species	Conidiation	Pathogenicity	References
MoATG1	*Magnaporthe oryzae*	Reduced	Lost	Liu et al. (2007a)
UsATG1	*Ustilago maydis*	Reduced	Reduced	Nadal and Gold (2010)
FgATG1	*Fusarium graminearum*	Reduced	Reduced	Lv et al. (2017)
BcATG1	*Botrytis cinerea*	Reduced	Lost	Ren et al. (2017)
BbATG1	*Beauveria bassiana*	Reduced	Reduced	Ying et al. (2016)
AoATG1	*Aspergillus oryzae*	Reduced	Not mentioned	Yanagisawa et al. (2013)
MrATG1	*Metarhizium robertsii*	Reduced	Reduced	Duan et al. (2013)
AfATG1	*Aspergillus fumigatus*	Reduced	Normal	Richie et al. (2007)
MoATG13	*Magnaporthe oryzae*	Normal	Normal	Dong et al. (2009)
AoATG13	*Aspergillus oryzae*	Reduced	Not mentioned	Kikuma and Kitamoto (2011)
MoATG17	*Magnaporthe oryzae*	Reduced	Lost	Kershaw and Talbot (2009)
FgATG17	*Fusarium graminearum*	Normal	Normal	Lv et al. (2017)
AnATG17	*Aspergillus niger*	Normal	Not mentioned	Nitsche et al. (2013)

### Functions of Atg1 in fungi

Recent studies in *M. oryzae* have shown that autophagy is critical for the pathogenicity of rice blast fungus (Lu et al., 2009; Liu et al., 2015, 2017; Cheng et al., 2018; Zhu et al., 2018; Shi et al., 2019; Yin et al., 2019; Sun et al., 2022). MoAtg1 is closely associated with conidial germination, lipid droplet accumulation, appressorium turgor pressure, and pathogenicity. Deletion of *MoATG1* affects the autophagic process of *M. oryzae*, which make it unable to tolerate starvation. All of these defects cause the Δ*Moatg1* mutants to be non-pathogenic (Liu et al., 2007b). UmAtg1 plays a critical role in autophagy and pathogenic development in *Ustilago maydis*, and deletion of *UmATG1* causes a decrease in the budding of haploid sporidia and affects survival under conditions of prolonged nutritional starvation (Nadal and Gold, 2010). Knockdown of *FgATG1* causes a complete block of the autophagic process in *Fusarium graminearum*. And disruption of *FgATG1* slows colony growth, decreases conidiation, and significantly reduces the percentage of diseased spikes compared to the wild type (Lv et al., 2017). Disruption of *BcATG1* impairs the autophagic process in *Botrytis cinerea* by inhibiting the accumulation of autophagosomes in the vacuoles. BcAtg1 is involved in conidia, sclerotial formation, lipid droplet accumulation in conidia, and trophic growth processes. The ability of Δ*Bcatg1* mutants to form appressorium infection structures is also diminished. Consistent with this result, Δ*Bcatg1* mutants are significantly less pathogenic to different host plant tissues compared to the wild type (Ren et al., 2017). PaAtg1 plays a key role in autophagy in *Podospora anserina*. Knockdown of *PaATG*1 completely abolishes autophagy and does not result in autophagosome formation. Compared to the wild type, Δ*Paatg1* mutants exhibit slightly slower nutritional growth, sparser aerial mycelium, and a lighter mycelium color. Δ*Paatg1* mutants also do not produce protoperithecia, which results in female sterility (Pinan-Lucarre et al., 2005).

BbAtg1 is essential for autophagy in *Beauveria bassiana*. Transmission electron microscopy images revealed no autophagosomes in the vacuoles of Δ*Bbatg1* mutants under nutrient starvation conditions. BbAtg1 is required for fungal conidiation, blastospore development, and full virulence (Ying et al., 2016). AoAtg1 is involved in the primary phase of autophagy induction in *Aspergillus oryzae*. AoAtg1 also plays an important role in the Cvt pathway. Under nitrogen starvation conditions, deletion of *AoATG1* lead to defects in aerial hyphal growth, conidiation, and conidial germination (Yanagisawa et al., 2013). The Δ*Mratg1* mutants lead to defects in aerial hyphal growth, conidiation, and fungal virulence in *Metarhizium robertsii* (Duan et al., 2013).

Δ*Afatg1* mutants disrupt autophagy and reduce conidiation in *Aspergillus fumigatus* (a human pathogen) due to abnormal conidiophore formation. However, the autophagic disruption caused by deletion of *AfATG1* has no significant effect on virulence in a mouse model. These results suggest that the pathogen does not require AfAtg1 for growth in the host (Richie et al., 2007). Atg1 has the same or different functions in different fungi. We found that in plant pathogenic fungi (*M. oryzae*, *U. maydis*, *F. graminearum*, *B. cinerea*, and *P. anserina*) and insect pathogenic fungi (*B. bassiana*, *A. oryzae*, and *M. robertsii*), deletion of *ATG1* impaired autophagy, which reduced pathogenicity to the host. Autophagy is impaired due to *ATG1* deletion, which has an important function in production applications. A recent study in *Penicillium chrysogenum* has found that autophagy is blocked due to the absence of *ATG1*, which increased the production of the β-lactam antibiotic penicillin (PEN; Bartoszewska et al., 2011).

### Functions of Atg13 and Atg17 in fungi

Although the Atg1-Atg13-Atg17 complex plays a key role in the initiation of autophagy in eukaryotes, studies of Atg13 and Atg17 functions in other fungi exist rarely. Atg13 and Atg17 are crucial for autophagy in *S. cerevisiae*. ∆*atg13* mutants fail to induce autophagy due to the inability to form the Atg1 kinase complex. ∆*atg17* mutants produce autophagosomes in less than half the normal size under starvation conditions and are severely impaired in the autophagic process (Cheong et al., 2005; Kabeya et al., 2005). In contrast, AoAtg13 is not required for autophagy of *A. oryzae* (Kikuma and Kitamoto, 2011). ∆*Fgatg17* mutants in *F. graminearum* have no effect on the deoxynivalenol production and pathogenicity of wheat, which indicate that the mutants may have a non-essential function in autophagy. Consistent with this finding, GFP-FgAtg8 protein degradation was not affected in the absence of *FgATG17* in *F. graminearum* (Lv et al., 2017). Knockdown of *AnATG17* in *Aspergillus niger* did not differ significantly from the wild type, except for a slight reduction in spore formation. ∆*Anatg17* mutants have little or no influence on the autophagic process in *A. niger* (Nitsche et al., 2013).

## Conclusion and perspectives

The molecular events of autophagy initiation are elusive in the autophagy process. We establish a structural framework that interprets how the Atg1/ULK1 complex is involved in the earliest steps in phagophore biogenesis. The Atg1/ULK1 complex is an important hub that integrates upstream nutritional signals *via* different signaling pathways and regulates different downstream substrates to participate in multiple steps of autophagy. TORC1 inhibits autophagy in response to nutrient-rich conditions, and AMPK promotes autophagy. Both upstream regulators directly control Atg1/ULK1 activity to mediate autophagy induction. TOR and AMPK are directly phosphorylated by Atg1/ULK1, which provides an additional level of regulatory feedback to modulate and fine-tune autophagy (Alers et al., 2012; Table 2). A range of Atg1/ULK1 substrates are explored recently in yeast and mammals. Researchers have found that Atg1/ULK1-dependent phosphorylation of other components of the pathway is involved in multiple steps of autophagy in proper temporal and spatial cues (Table 3). The functional significance of Atg1 phosphorylation is gradually clear for Atg4, Atg9, Ykt6, Atg11, and Atg1. The crucial roles of ULK1 phosphorylation in the ULK1 complex (Atg13, FIP200, and Atg101), Atg9 and PI3KC3-C1 subunits (beclin-1, Vps34/PIK3C3, and AMBRA1) are preliminarily interpreted. Our expanded understanding of Atg1/ULK1 regulation by mTORC1 and AMPK raises the question of how the kinase is differentially activated by nutrients or selective cargoes. And whether intermolecular interactions of the Atg1/ULK1 complex affect the conformation of the Atg1 kinase domain and thereby control its activity? The upstream and downstream regulation mechanism of Atg1 complex has been well studied in yeast and mammals, and the autophagy functions of the Atg1 complex in pathogenic fungi have been gradually revealed. However, the molecular regulation mechanism of Atg1 complex in autophagy of plant pathogenic fungi remains to be studied. *M. oryzae* is a model organism for studying the autophagy of filamentous fungi. A key prerequisite for the initiation of autophagy is the precise regulation of the MoAtg1 complex. What upstream components regulate the Atg1 complex? What substrates are necessary for autophagy in the MoAtg1 complex? What specific steps are involved in the regulation of substrates by MoAtg1 complex in autophagy? Previous studies have reported that there is only one PAS site in *S. cerevisiae*, but there are multiple PAS sites in *M. oryzae* (Dong et al., 2009). How is the MoAtg1 complex assembled at the PAS sites? And how does MoAtg1 complex recruit downstream autophagy proteins at the PAS sites? PAS sites play a key role in the autophagosome formation, and thus the role of MoAtg1 complex in the autophagosome formation deserves further investigation.

**Table 2:** The Atg1/ULK1 complex is phosphorylated by upstream kinases TOR and AMPK.

Species	Protein	Residue	Kinase	Functional effect	References
*S. cerevisiae*	Atg13	Ser496	TOR	Required for autophagy induction	Fujioka et al. (2014)
	Atg1	Thr226 and Ser230	TOR	Required for Atg1 kinase activity	Kijanska et al. (2010)
Mammals	ULK1	Ser758	mTORC1	Prevents ULK1 from interacting with AMPK	Kim et al. (2011), Shang et al. (2011)
		Ser638	mTORC1 and AMPK	Facilitates Phosphorylation at Serine 758 and Proper	Shang et al. (2011)
		Ser467, Ser555, Thr574 and Ser637	AMPK	Required for autophagy and mitophagy	Gwinn et al. (2008)
		Ser757	mTORC1	Promotes s the interaction between ULK1 and AMPK	Kim et al. (2011), Shang et al. (2011)
	TSC2	Thr1227 and Ser1345	AMPK	Activates autophagy	Inoki et al. (2003)
	mTOR binding partner raptor	Ser722 and Ser792	AMPK	Activates autophagy	Gwinn et al. (2008)

**Table 3:** The Atg1/ULK1 kinase phosphorylates downstream substrates.

Species	Protein	Residue	Kinase	Functional effect	References
*S. cerevisiae*	Atg2	Ser249 and Ser1086	Atg1	Have no impact on autophagy	Papinski et al. (2014)
	Atg1	Thr226 and Ser230	Atg1	Activates Atg1 kinase	Yeh et al. (2010)
	Atg4	Ser307	Atg1	Protects the synthesis and maintenance of the Atg8-PE pool	Sanchez-Wandelmer et al. (2017)
	Ykt6	Ser182 and Ser183	Atg1	Participates in the fusion of autophagosomes and vacuoles	Barz et al. (2020)
	Atg11	Ser949, Ser1057 and Ser1064	Atg1	Participates in the regulation of selective autophagy	Liu et al. (2012)
Mammals	Atg4B	Ser316	ULK1	Protects the synthesis and maintenance of the Atg8-PE pool	Pengo et al. (2017)
	FIP200	Ser943, Ser986 and Ser1323	ULK1	Unkonwn	Egan et al. (2015)
	Atg101	Ser11 and Ser203	ULK1	Unkonwn	Egan et al. (2015)
	Beclin-1	Ser14	ULK1	Required for autophagy induction	Russell et al. (2013)
	AMBRA1	Ser465 and Ser635	ULK1	Required for autophagy induction	Egan et al. (2015)
	Vps34	Ser249	ULK1	Have no impact on autophagy induction	Egan et al. (2015)

Although the autophagy pathway is a conserved process from yeast to mammals, there are some differences in the composition of the Atg1 complex between *M. oryzae* and yeast/mammals. In *M. oryzae*, the MoAtg1 complex consists of MoAtg1 (Liu et al., 2007b), MoAtg13, MoAtg17, MoAtg29, and MoAtg101 (data not published). In addition to Atg1-Atg13-Atg17/FIP200, Atg1 complex of *S. cerevisiae* also contains Atg29 and Atg31, and Atg1/ULK1 complex of *S. pombe* and mammals also contains a unique Atg101 subunit. The MoAtg1 complex composition of *M. oryzae* is different from that of yeast and mammals. The functions of MoAtg29 and MoAtg101 are not clear. The effects of MoAtg29 and MoAtg101 on pathogenicity of *M. oryzae* are not clear? Additionally, it is unclear how MoAtg29 and MoAtg101 are put together in the MoAtg1 complex? Atg29 and Atg31 can stabilize and maintain Atg17 curvature in *S. cerevisiae* (Chew et al., 2013). In *S. pombe* and mammals, Atg101 has the function of stabilizing the Atg13 HORMA domain (Hegedus et al., 2014; Michel et al., 2015; Qi et al., 2015; Suzuki H. et al., 2015). Besides, the unique Atg101 subunit may have other important functions due to its ability to interact with the CTD domain of Atg1 (Nanji et al., 2017). These studies provide theoretical basis for the role of MoAtg29 and MoAtg101 and the assembly of MoAtg1 complex in *M. oryzae*. The homolog of *S. cerevisia*e Atg31 has been not found in *M. oryzae*, indicating that the MoAtg17-MoAtg29-MoAtg31 complex is not conserved in the evolutionary process. Here, we hypothesize that MoAtg101 replaces the function of Atg31 of *S. cerevisiae* in *M. oryzae*, and that MoAtg101 and MoAtg29 are involved in maintaining the curvature of MoAtg17. This hypothesis will need to be verified by further investigation. In addition, the Atg1-Atg13 complex is conserved from yeast to mammals and is essential for autophagy in yeast. Atg13 has also been reported to be involved in autophagy in other species (Chan et al., 2009; Chang and Neufeld, 2009; Ganley et al., 2009; Hosokawa et al., 2009; Jung et al., 2009). However, the *M. oryzae* Δ*Moatg13* mutant has no effect on the pathogenicity of the host plant, which is similar to the wild-type Guy11 phenotype. In addition, DsRed2-MoAtg8 and EGFP-MoAtg9 expression profiles of Δ*Moatg13* mutants are similar to those of Δ*MoAtg9* mutants. We propose a hypothesis that MoAtg13 loses its autophagy function in *M. oryzae* and the MoAtg1-MoAtg13 complex is not preserved during evolution process. Furthermore, some proteins have more than one function and are involved in crosstalk between autophagy and other signaling pathways (Li et al., 2017; Zheng et al., 2017, 2018; Zhu et al., 2018). Although the assembly of Atg1/ULK1 complex in yeast and mammals has been clearly studied, there is little research in pathogenic fungi. In *M. oryzae*, we used Alphafold2 to predict the protein structure of MoAtg1, MoAtg17, and MoAtg101. And we preliminarily explore that MoAtg1, MoAtg13, and MoAtg17 interact each other and that MoAtg101 interacts with MoAtg13 by yeast two-hybrid methods (data not published). These studies lay the foundation for studying how the MoAtg1 complex is assembled in *M. oryzae* and lead to further systematic studies on the evolution of Atg1 initiation complex in eukaryotes.

The precise regulation of autophagy by pathogenic fungi at different stages plays an important role in the process of plant-pathogen interaction, especially in the process of pathogen invasion. The MoAtg1 complex is essential for pathogen invasion into living host tissues, as in *M. oryzae*, the Δ*Moatg1* mutants prevent appressorium from producing infected mycelia on host tissues (Liu et al., 2007b). However, the role of the MoAtg1 complex in host-pathogen expansion remains unknown. Currently, drug targeting and protein crystallization approaches are used to study the assembly pattern, upstream regulatory factors, downstream recognition of substrates, and drug targets in MoAtg1 complex of *M. oryzae*. These findings will help us elucidate the processes and functions of plant-pathogen interactions and provide new approaches for crop disease control.

## Author contributions

Y-YC: conceptualization, investigation, formal analysis, figure preparations, and writing—review and editing. X-MZ and LL: conceptualization, formal analysis, figure preparations, and writing—review and editing. J-PL and X-HL: conceptualization, supervision, and writing—review and editing. F-CL: project administration, resources, supervision, and writing—review and editing. All authors contributed to the article and approved the submitted version.

## Funding

This study is supported by grants from the National Natural Science Foundation of China (31970140, 32100159, and 31972216), by grants from the Special Project for the Selection and Breeding of New Agricultural Varieties in Zhejiang Province, China (2021C02064), by grants from the Key Research and Development Project of Zhejiang Province, China (2021C02010), and by a grant Organism Interaction from Zhejiang Xianghu Laboratory to F-CL.

## Conflict of interest

The authors declare that the research was conducted in the absence of any commercial or financial relationships that could be construed as a potential conflict of interest.

## Publisher’s note

All claims expressed in this article are solely those of the authors and do not necessarily represent those of their affiliated organizations, or those of the publisher, the editors and the reviewers. Any product that may be evaluated in this article, or claim that may be made by its manufacturer, is not guaranteed or endorsed by the publisher.
